# Changes in the Swine Gut Microbiota in Response to Porcine Epidemic Diarrhea Infection

**DOI:** 10.1264/jsme2.ME15046

**Published:** 2015-07-25

**Authors:** Hyeon-Woo Koh, Myun Soo Kim, Jong-Soo Lee, Hongik Kim, Soo-Je Park

**Affiliations:** 1 Department of Biology, Jeju National University, Jejudaehak-ro 102, Jeju 690–756, Republic of Korea; 2 R&D Division, VITABIO, Inc., Daejeon 300–728, Republic of Korea; 3 College of Veterinary Medicine (BK21 Plus Program), Chungnam National University, Daejeon 305–764, Republic of Korea

**Keywords:** Porcine epidemic diarrhea, microbiota, 16S rRNA gene, large intestine, next-generation sequencing

## Abstract

The gastrointestinal tract of mammals is a complex ecosystem with distinct environments and comprises hundreds of different types of bacterial cells. The gut microbiota may play a critical role in the gut health of the host. We herein attempted to identify a microbiota shift that may be affected by porcine epidemic diarrhea (PED). We observed significant differences in microbiota between the control and PED virus (PEDV)-infected groups at both the phylum and genus level. Most commensal bacteria (*i.e. Psychrobacter*, *Prevotella*, and *Faecalibacterium*) in the healthy gastrointestinal tract were decreased due to dysbiosis induced by PEDV infection.

Porcine epidemic diarrhea (PED) is an enteric disease in swine caused by PED virus (PEDV), which is a member of the family *Coronaviridae* (21). PEDV was first identified in England in 1971 (17), and outbreaks of the virus have since been reported and recently in Europe, Asia, and the USA (4, 20, 25, 27). The first case of a virus outbreak occurred in South Korea in 1992, and has since been followed by yearly outbreaks (11, 24). PED poses the most serious threat to infant piglets, with morbidity and mortality rates previously reported to be between 80 and 100% (19), thereby engendering economics losses in the pork industry.

The gut microbiota is a complex consortium of 10–100 trillion microbial cells in the gastrointestinal tract and influences many aspects of its host (31). Numerous animal studies, including those on mammals, noted the importance of the microbiota for the function and health of the gastrointestinal (GI) tract (8, 18, 29). The GI microbiota at its mature stage is generally considered to be stable with minimal changes in its composition; however, the microbial community is subject to change due to factors including diet, age, or health (13). Due to physiological, nutritional, and immunological contributions of the GI microbiota to the gut health of the host, the structure and functional roles of the biota need to be examined in more detail. We previously reported that some microorganisms were associated with swine health and development (18). Previous studies on PED have mainly focused on sequence-based phylogeny analyses, pathogenicity, treatments, and/or vaccinations, without investigating the relationship between the GI microbiota and PEDV infection. In order to address this critical gap, we herein examined specific microorganisms and/or microbial compositions from fecal samples collected from the large intestine, and compared healthy swine and PEDV infected-swine groups using Illumina MiSeq high-throughput sequencing.

Fourteen fecal samples were collected from two different swine groups: healthy (labeled as normal, *n*=7) and PEDV infection-diagnosed (designated as infected, *n*=7) pigs. Pigs were Yorkshire-Landrace-Duroc crossbred pigs from a live-stock farmhouse located in South Korea, and were less than 3 months old at the time of collection. PEDV-infected swine were diagnosed with the infection at the College of Veterinary Medicine at Chungnam National University between 2013 and 2014. All fecal samples were collected from the large intestine and were immediately placed into sterile conical tubes using alcohol-sterilized spatulas, and were then stored at −80°C until later analysis. DNA extraction, a polymerase chain reaction for paired-end sequencing performance, and bioinformatics analysis were described previously (9, 18). Sequencing was performed by the Beijing Genomics Institute (Hong Kong, China) using the MiSeq system (Illumina, San Diego, CA, USA), following the system manufacturer’s instructions. In the bioinformatics analysis, we used modified pipelines as described on the websites: the mothur project (http://www.mothur.org/wiki/MiSeq_SOP) (23) and Quantitative Insights into Microbial Ecology (QIIME) (http://qiime.org/tutorials/illumina_overview_tutorial.html) (3). The bacterial sequence reads were compared with known 16S rRNA genes as reference data obtained from the Ribosomal Database Project (RDP), and were taxonomically assigned based on RDP classifiers (5).

Table 1 summarizes the sequencing reads, diversity indices, and sample coverages of the swine gut samples. After quality control processing and chimeric removal, this study used a total of *ca.* 600,000 reads (from 646,705 raw reads) to analyze abundance and diversity, as well as to conduct a taxonomic comparison. We used an unweighted pair group method with arithmetic mean clustering (UPGMA) and nonmetric multidimensional scaling (NMDS) analyses, and observed the clear separation of the bacterial community structures of the swine gut into two groups (Fig. 1 and Fig. S1). We also conducted a pairwise comparison analysis of similarity distances of bacterial communities, and the results were consistent with our previous findings obtained using the UPGMA and NMDS method. In the comparison analysis, the gut microbiota of the infected group showed higher similarities than that of the normal group (Fig. S2). According to our measurement of OTUs, Shannon, Chao, and Simpson indices, the gut microbiota diversity of the normal group was higher than that that of the infected group (*P*<0.05, Table 1). This result was inconsistent with our previous findings, in which the diversity indices for the gut microbiota of a runt swine group and healthy group were higher (18). Using these results, we hypothesized that the diarrhea events of the host caused by PEDV infection may remove or “sweep off” most microorganisms in the gut of the host. PEDV infection-induced dysbiosis may leave host swine afflicted with a range of pathogenic agents.

We observed bacterial communities including typical intestinal bacterial groups in our samples, with the dominant ones being phyla *Firmicutes*, *Bacteroidetes*, *Proteobacteria*, and *Fusobacteria*, which is consistent with previous findings on swine gut microbiota (8, 10, 14, 18). In the normal group, the phylum *Firmicutes* marked the highest number of individuals among all microorganisms (approximately 57% of total reads), followed by *Proteobacteria* (25.1%), *Bacteroidetes* (10.4%), unclassified *Bacteria* (3.7%), *Actinobacteria* (3.2%), and *Spirochaetes* (0.9%) (Fig. S3). In contrast, the phylum *Fusobacteria* was dominant in the infected group (approximately 32%, Fig. S3, *cf.* 0.1% in the normal group). This was interesting because *Fusobacteria* spp. are typically dominant in the mouth as obligate anaerobic and non-spore-forming bacteria. *Fusobacteria* spp. have also been frequently identified in a wide variety of clinically significant anaerobic infection cases with other anaerobes, including oral and dental infections (*e.g.* dental caries), brain abscesses, and tissue infections (2). We gained a more detailed insight by examining results at the phylum and genus levels. At the phylum level, the normal group and PEDV-infected group both had dominance members. However, a difference was detected between the two groups due to a microbial imbalance (*i.e.* dysbiosis) in the infected group only (28). We also compared genus level taxonomic resolutions between the normal and infected groups. In order to achieve this, we analyzed more than 1% of the total assigned reads and relatively re-calculated the proportions of taxonomic assigned reads (Fig. 2). As expected, a difference was observed in the occurrence and abundance of identified genera between the normal and infected groups. *Psychrobacter* (45.3%) and *Lactobacillus* (25.8%) were the most abundant genera in the normal group. *Prevotella*, *Carnobacterium*, *Faecalibacterium*, *Sporosarcina*, *Paenibacillus*, and *Clostridium* genera in that order of abundance were also identified in the normal group. On the other hand, in the infected group, eight genera were more abundant in the gut, including *Fusobacterium* (30.3%) and *Bacteroides* (26.9%). With the exception of the genera *Psychrobacter* and *Carnobacterium*, the abundances of the genera *Fusobacterium*, *Escherichia* (*Shigella*), *Prevotella*, and *Faecalibacterium* significantly differed (*P*<0.05) between the normal and infected groups. The abundances of pathogenic organisms including *Fusobacterium* and *Escherichia* (*Shigella*) were markedly increased in the infected group (1, 6), while most commensal bacteria (*i.e. Psychrobacter*, *Prevotella*, and *Faecalibacterium*) observed in the healthy gastrointestinal tract decreased. This may have serious implications because these commensal bacteria have been suggested to play important roles such as active digestive enzyme- and/or as short chain fatty acid-producing bacteria. Previous studies indicated that commensal bacteria were crucial for gut health and had functions against diseases including rheumatoid arthritis, ulcerative colitis, and autism (7, 15, 16, 22). One of the commensal bacteria, *Psychrobacter* spp., as a probiotic, may improve the diversity of the autochthonous gut microbiota by stimulating its growth (26, 30). Furthermore, most species of the genus *Carnobacterium* function as producers of lactic acid through carbohydrate fermentation and carnobacterium bacteriocins. Moreover, a previous study reported that they had ability to inhibit the growth of *Listeria monocytogenes* (*i.e.* food protection) (references therein; 12).

In order to unravel gut microbiota interactions between normal and infected groups, we explored and constructed weighted-OTUs networks. One assumption of this study was that each microorganism (*i.e.* OTUs) formed a set of consortium within each group, which was proven to be correct by an examination of the profiles of UPGMA, NMDS, and similarity analysis of the gut microbiota (Fig. S4). We observed similarities between the weighted-OTUs network results and other analyses (*e.g.* NMDS, UPGMA); most OTUs were clearly separated in accord with the group. However, other OTUs were shared within other groups. The latter OTUs are considered common taxa such as *Firmicutes* and *Bacteroides*.

This study successfully showed viral infection-induced dysbiosis in the GI tract. Our results substantiate the potential link between the balance of microorganisms in the GI and swine health and development. Future studies are needed in order to determine the functional relationship between diarrhea-causing agents including PEDV and dysbiosis/recovery of the gut microbiota.

The nucleotide sequences obtained in this study were deposited at the EMBL-EBI European Nucleotide Archive under the study accession number PRJEB8875.

## Supplementary Information



## Figures and Tables

**Fig. 1:**
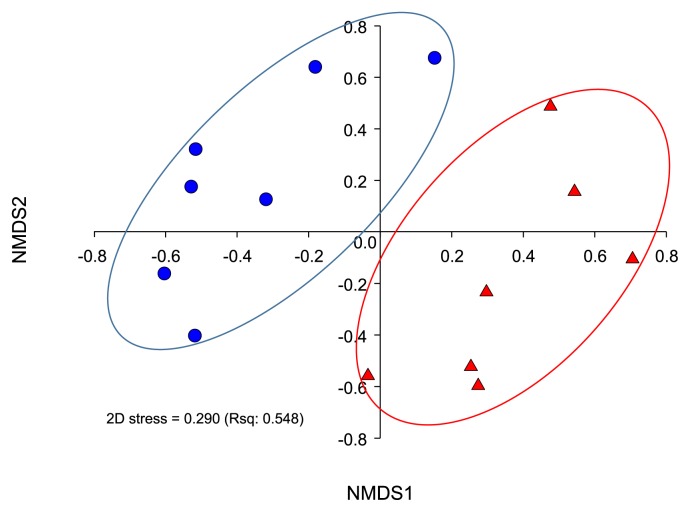
Relationships between gut microbiota profiles of samples, represented by a nonmetric multidimensional scaling (NMDS) from the mothur package. Operational taxonomic units (OTUs) were determined based on 97% similarity in reads. PEDV-infected and normal samples are represented as different shapes and colors: triangle (red) and circle (blue), respectively.

**Fig. 2:**
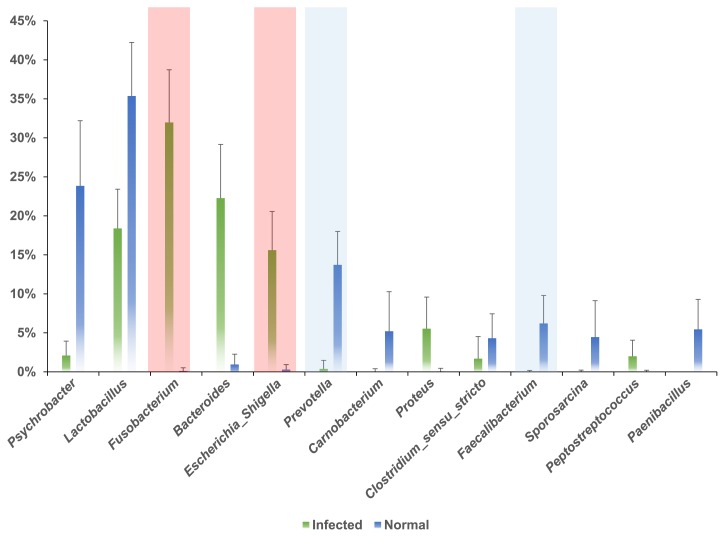
Abundances of the indicated genera in normal and PEDV-infected gut samples. Bacterial 16S rRNA gene sequences were assigned to each genus using the mothur package and a reference database of known 16S rRNA genes obtained from the Ribosomal Database Project. The green closed bar and blue closed bar indicate the infected and normal groups, respectively. The values of genera abundances for each group were calculated by an arithmetic average. The error bars indicate standard errors of the average. Red (represented as the infected group) and blue (represented as the normal group) box indicate significant differences (*P*<0.05).

**Table 1:** Description of gut microbiota from the large intestinal tract of swine and estimates of sequence diversity and phylotype coverage of MiSeq data. Diversity indices and richness estimators were calculated using the mothur package (of the mother project; http://www.mothur.org). Diversity was estimated using operational taxonomic units (OTUs) and defined as groups with ≥97% sequence similarity. All pigs determined no medical records.

Sample	Group	Gender*	Total reads	S & Q** reads	Analyzed reads	Observed OTUs	Shannon	Chao	Simpson	Good’s coverage
ped1_16	Infected	F	97,277	45,796	44,817	438	2.249	1116.276	4.942	0.994
ped1_18	Infected	M	64,239	30,262	29,743	504	2.456	890.288	5.165	0.993
ped1_19	Infected	M	98,613	46,187	45,435	514	2.552	721.030	6.118	0.995
ped1_6	Infected	F	116,735	54,949	53,016	315	1.841	1713.158	3.311	0.996
ped1_7	Infected	F	63,211	29,599	28,449	331	2.433	988.027	6.681	0.992
ped1_8	Infected	M	102,990	48,674	47,758	349	2.044	954.800	4.625	0.995
ped1_9	Infected	F	86,115	40,476	39,855	301	2.149	844.750	3.116	0.996
ped2_12	Normal	M	111,101	50,842	48,451	1106	4.585	2004.091	29.401	0.991
ped2_14	Normal	M	112,492	52,925	42,505	990	4.486	1957.653	34.713	0.990
ped2_16	Normal	M	150,109	70,865	69,907	549	0.618	1386.545	1.187	0.995
ped2_18	Normal	F	120,862	56,646	43,360	937	3.579	1947.683	10.213	0.989
ped2_2	Normal	F	78,684	37,220	33,552	694	3.575	1614.957	13.270	0.989
ped2_20	Normal	M	97,828	46,219	39,435	916	4.237	1968.683	20.823	0.989
ped2_8	Normal	F	76,586	36,045	33,376	730	3.964	1231.774	18.014	0.991

* F and M denote female and castrated male swine, respectively.

** S & Q denote “subsampled” and “qualified”, respectively.
