# Supplementary material for: Changes in the Swine Gut Microbiota in Response to Porcine Epidemic Diarrhea Infection
Source: Microbes Environ. 2015 Jul 25;30(3):284–7. doi: 10.1264/jsme2.ME15046 (PMC4567570; doi:10.1264/jsme2.ME15046)
Supplement: Supplementary file 1 [file 30_284_s1.pdf]

Supplementary materials

PEDV detection

To detect PEDV, all rectal swab samples were collected from 7 piglets infected with PEDV from December 2013 to December 2014. These infected piglets were selected from the one swine farms located in South Korea. The rectal swabs were soaked in PBS buffer supplemented with penicillin G (10,000 IU/mL) and streptomycin (2 mg/mL) and centrifuged at 2,000 ×g for 15 min to collect the supernatant fluids. The supernatant fluids were stored at –80°C until used. For RNA extraction, we used TRIzol Reagent (Invitrogen, Carlsbad, CA, USA) following the manufacturer's instructions. Briefly, the supernatant (200 µL) containing PEDV was mixed with 1 mL of TRIzol Reagent. Then, 200 µL of chloroform was added to the mixture, and the suspension was centrifuged for 15 min at 10,000 ×g. The RNA-containing aqueous phase was precipitated with the same volume isopropanol, maintained at –20°C for 1 h, and centrifuged for 15 min at 10,000 ×g. The resulted RNA pellet was washed with 1 mL of 75% (v/v) absolute ethanol, centrifuged for 15 min at 10,000 ×g and dried, and resuspended in 30 µL of nuclease-free water. We used the specific primer set for N (nucleocapsid) and M (membrane protein) genes (1, 2). RT-PCR was performed using iScript^{TM} One-Step RT-PCR Kit with SYBR® Geen (BioRad) following the manufacturer's instructions. RT-PCR products were visualized via electrophoresis in a 1~2% (w/v) agarose gel containing ethidium bromide. Bands of the correct size were estimated on agarose gel (Fig. S5).

Supplementary figure legends

Fig. S1. An unweighted pair group method with arithmetic mean (UPGMA) clustering tree. Operational taxonomic units (OTUs) were determined based on 97% similarity of reads. Blue and red coloring represent the normal and PEDV infected groups, respectively.

Fig. S2. Similarity distance for bacterial communities between normal and infected group in the swine gut (operational taxonomic unit [OTU] distance = 0.03). The color key indicates the level of similarity between the two groups. The normal and PEDV infected groups are represented by red and blue triangle, respectively.

Fig. S3. The abundances of the indicated phyla in the normal and infected group of gut microbiota. The bacterial 16S rRNA gene sequences were assigned to each phylum using the mothur package and a reference database of known 16S rRNA genes obtained from the Ribosomal Database Project. Inner and outer circle show PEDV infected group and normal group, respectively.

Fig. S4. OTU-based weights network reveals modules of OTUs associated with gut microbiota from normal and PEDV infected swine group. Each node represents one OTU and an edge is drawn between OTUs. OTUs were determined based on 97% similarity of reads. Blue, red, and green circle indicate PEDV infected, normal group, and OTU, respectively. Blue and red line denote OTU relationship for normal and PEDV infected swine group, respectively.

Fig. S5. Detection of the nucleocapside (a) and membrane protein (b) genes of the PEDV in specimens by RT-PCR. M denotes DNA ladder marker. Lanes 1–7: reaction with fecal specimens; lane (-): negative control. The arrowheads indicate the 854-bp fragment.

Fig. S1.

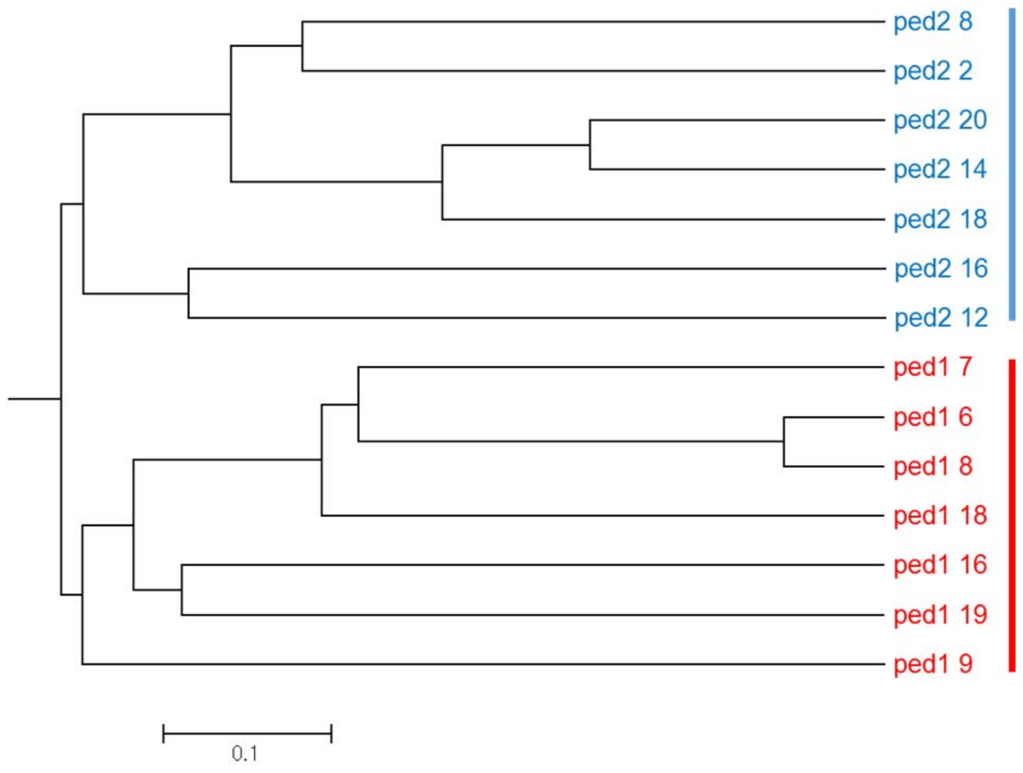
ped2 8
ped2 2
ped2 20
ped2 14
ped2 18
ped2 16
ped2 12
ped1 7
ped1 6
ped1 8
ped1 18
ped1 16
ped1 19
ped1 9
0.1

Fig. S2.

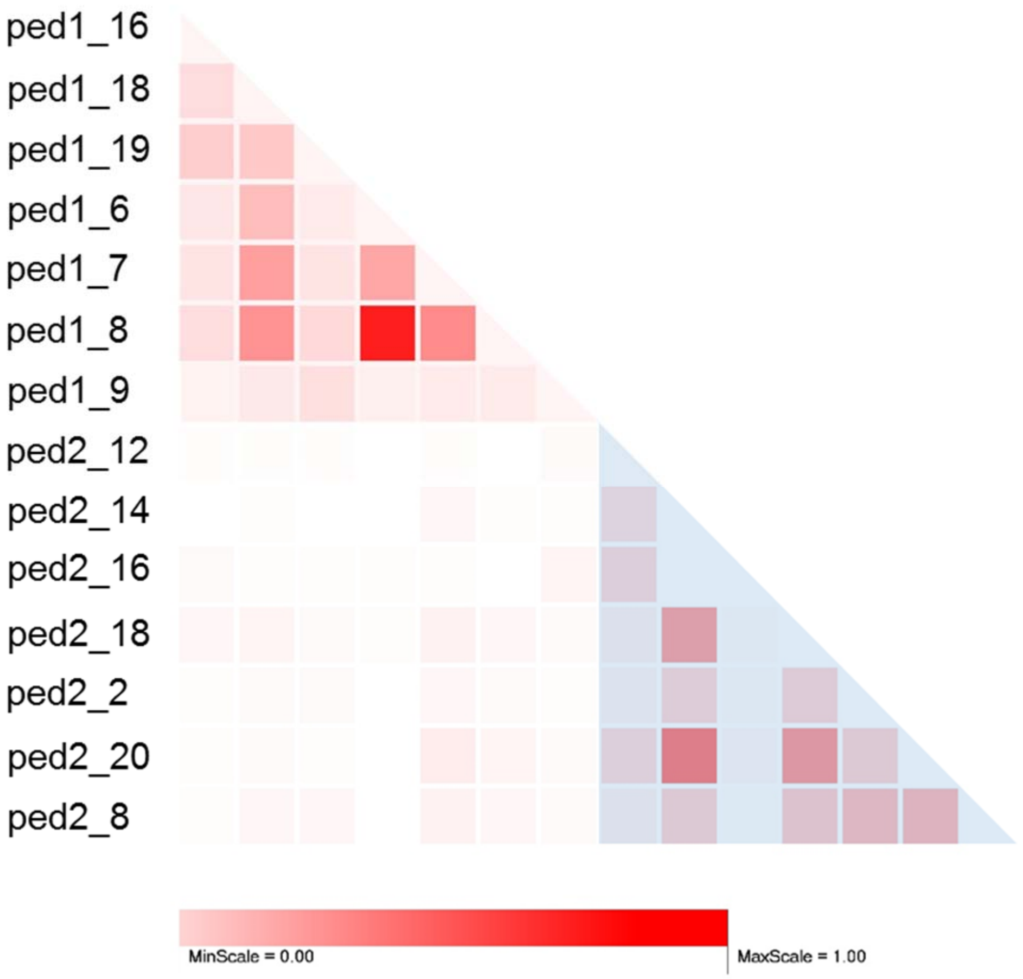
ped1_16
ped1_18
ped1_19
ped1_6
ped1_7
ped1_8
ped1_9
ped2_12
ped2_14
ped2_16
ped2_18
ped2_2
ped2_20
ped2_8
MinScale = 0.00
MaxScale = 1.00

Fig. S3.

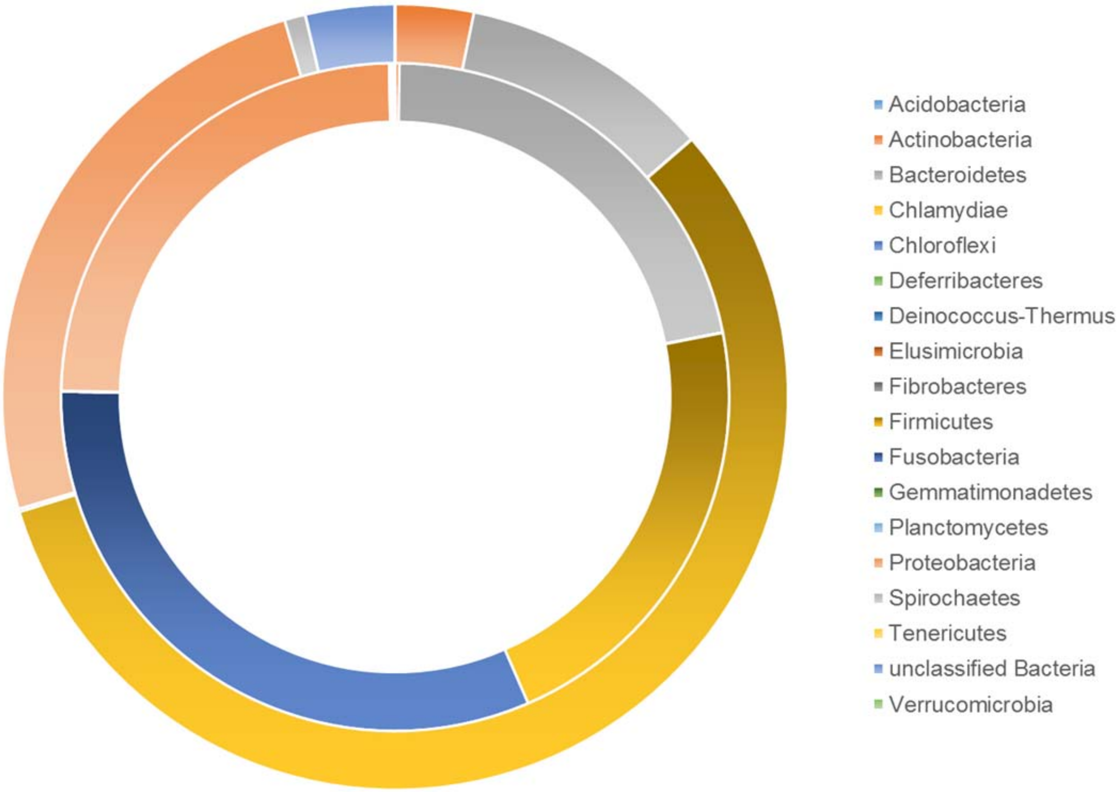
Acidobacteria
Actinobacteria
Bacteroidetes
Chlamydiae
Chloroflexi
Deferribacteres
Deinococcus-Thermus
Elusimicrobia
Fibrobacteres
Firmicutes
Fusobacteria
Gemmatimonadetes
Planctomycetes
Proteobacteria
Spirochaetes
Tenericutes
unclassified Bacteria
Verrucomicrobia

Fig. S4.

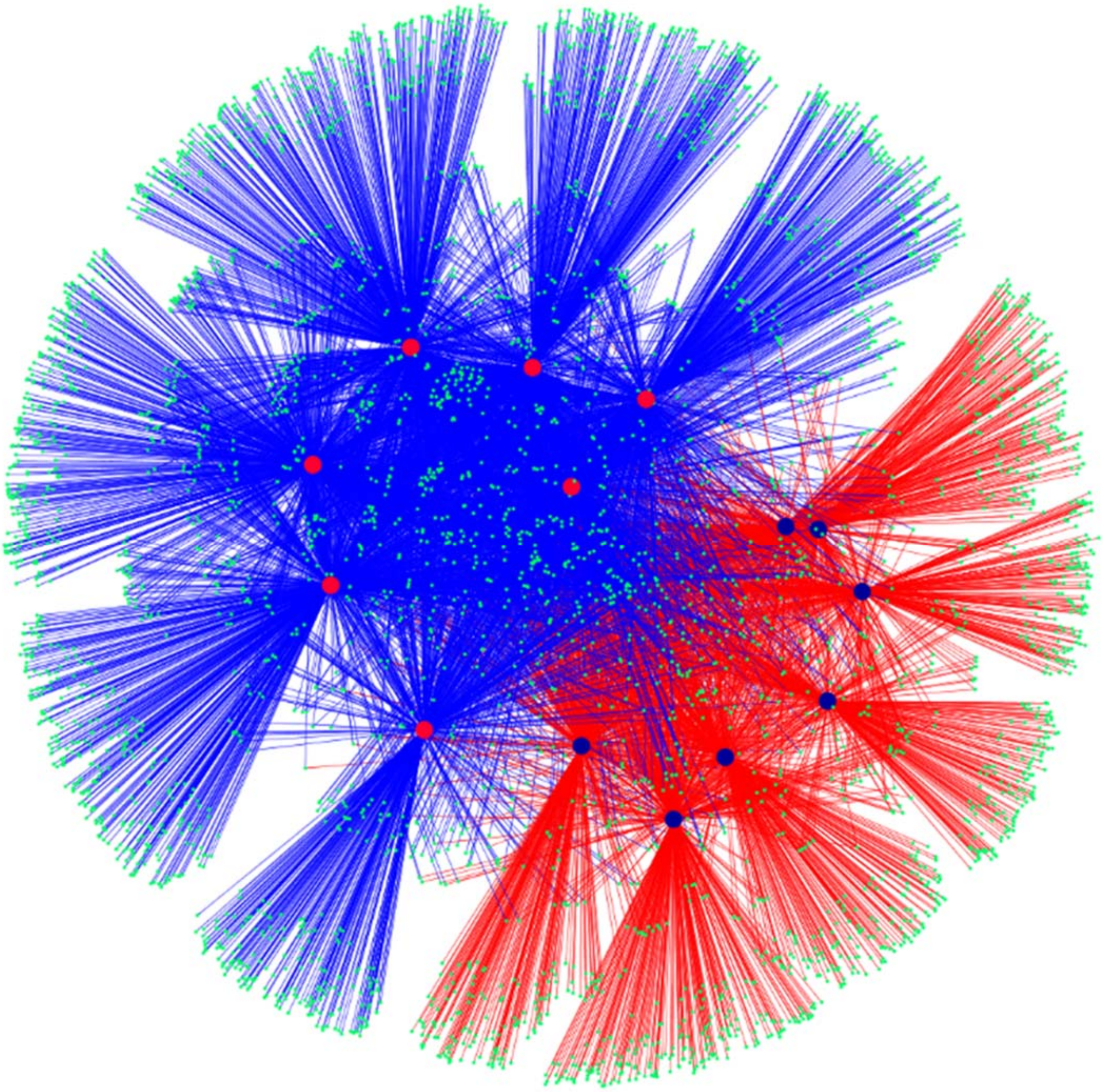

Fig. S5.

(a) Nucleocapside

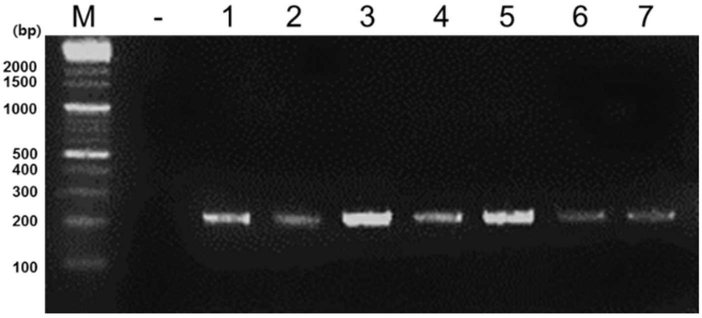

(b) Membrane protein

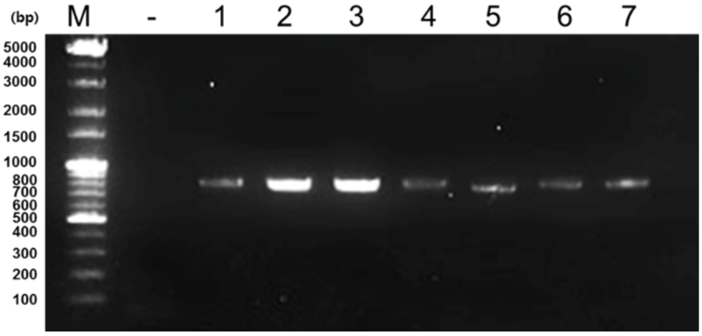

Supplementary references

1. Ishikawa, K., H. Sekiguchi, T. Ogino, and S. Suzuki. 1997. Direct and rapid detection of porcine epidemic diarrhea virus by RT-PCR. J. Virol. Methods. 69:191-195.

2. Kim, S.H., I.J. Kim, H.M. Pyo, D.S. Tark, J.Y. Song, and B.H. Hyun. 2007. Multiplex real-time RT-PCR for the simultaneous detection and quantification of transmissible gastroenteritis virus and porcine epidemic diarrhea virus. J. Virol. Methods. 146:172-177.
